
JOURNAL INFORMATION
==============================
NLM Title Abbreviation: Wirtschaftsdienst
Journal ID: Wirtschaftsdienst
NLM Journal ID: 101086612

Wirtschaftsdienst (Hamburg, Germany : 1949)

ISSN: 0043-6275

ARTICLE INFORMATION
==============================
PMCID: PMC7368605
PMID: 32834164
DOI: 10.1007/s10273-020-2690-7
Article ID: 2690
Article version: 1
Subjects: Zeitgespräch

Wirtschaftspolitik in der Corona-Krise: Stabilisierung muss durch eine kluge Transformationspolitik ergänzt werden

Fratzscher Marcel MFratzscher@diw.de

Michelsen Claus CMichelsen@diw.de

grid.8465.f 0000 0001 1931 3152 Deutsches Institut für Wirtschaftsforschung, Mohrenstr. 58, 10117 Berlin, Deutschland
Electronic publication date: 2020 Jul 18
Print publication date: 2020
Volume: 100
Issue: 7
First page: 484
Last page: 489
Copyright: © Der/die Autor(en) 2020
License: Open Access Dieser Artikel wird unter der Creative Commons Namensnennung 4.0 International Lizenz veröffentlicht, welche die Nutzung, Vervielfältigung, Bearbeitung, Verbreitung und Wiedergabe in jeglichem Medium und Format erlaubt, sofern Sie den/die ursprünglichen Autor(en) und die Quelle ordnungsgemäß nennen, einen Link zur Creative Commons Lizenz beifügen und angeben, ob Änderungen vorgenommen wurden.
Die in diesem Artikel enthaltenen Bilder und sonstiges Drittmaterial unterliegen ebenfalls der genannten Creative Commons Lizenz, sofern sich aus der Abbildungslegende nichts anderes ergibt. Sofern das betreffende Material nicht unter der genannten Creative Commons Lizenz steht und die betreffende Handlung nicht nach gesetzlichen Vorschriften erlaubt ist, ist für die oben aufgeführten Weiterverwendungen des Materials die Einwilligung des jeweiligen Rechteinhabers einzuholen.
Weitere Details zur Lizenz entnehmen Sie bitte der Lizenzinformation auf http://creativecommons.org/licenses/by/4.0/deed.de.
License URL: https://creativecommons.org/licenses/by/4.0/

issue-copyright-statement: © The Author(s) 2020

==============================

Die Corona-Pandemie hat die Weltwirtschaft in eine der schwersten Rezessionen seit der großen Depression der 1930er Jahre gestürzt. In den fortgeschrittenen Volkswirtschaften ist die Wirtschaftsleistung bereits im ersten Quartal dieses Jahres geschrumpft – ein Gros der Einschränkungen fällt in das zweite Quartal, sodass die Daten für den größten Rückgang der Wirtschaftsleistung weiterhin noch ausstehen. Auch in den Schwellenländern grassiert das Virus und beeinträchtigt das gesellschaftliche Leben erheblich. In einigen großen Volkswirtschaften kam es zuletzt zu einem zweiten, drastischen Anstieg der Infektionszahlen – allen voran in den USA. Die deutsche Wirtschaft wird ebenfalls kräftig durch die Krise erfasst und voraussichtlich die schärfste Rezession der Nachkriegsgeschichte erfahren.

Traf der wirtschaftliche Schock anfänglich hauptsächlich die Angebotsseite, so wird die wirtschaftliche Rezession zunehmend von der schwachen Nachfrage beim privaten Konsum und bei Investitionen getrieben. Die Produktionsstillstände führen zu Unterbrechungen in international verflochtenen Lieferbeziehungen. Kurzarbeit und Entlassungen drücken auf die Kaufkraft der privaten Haushalte und senken deren Nachfrage – massive Umsatzausfälle und die steigende Verschuldung schränken die Investiti-

Abbildung 1

**Medienindizes zur Verbreitung epidemiologischer Schocks**

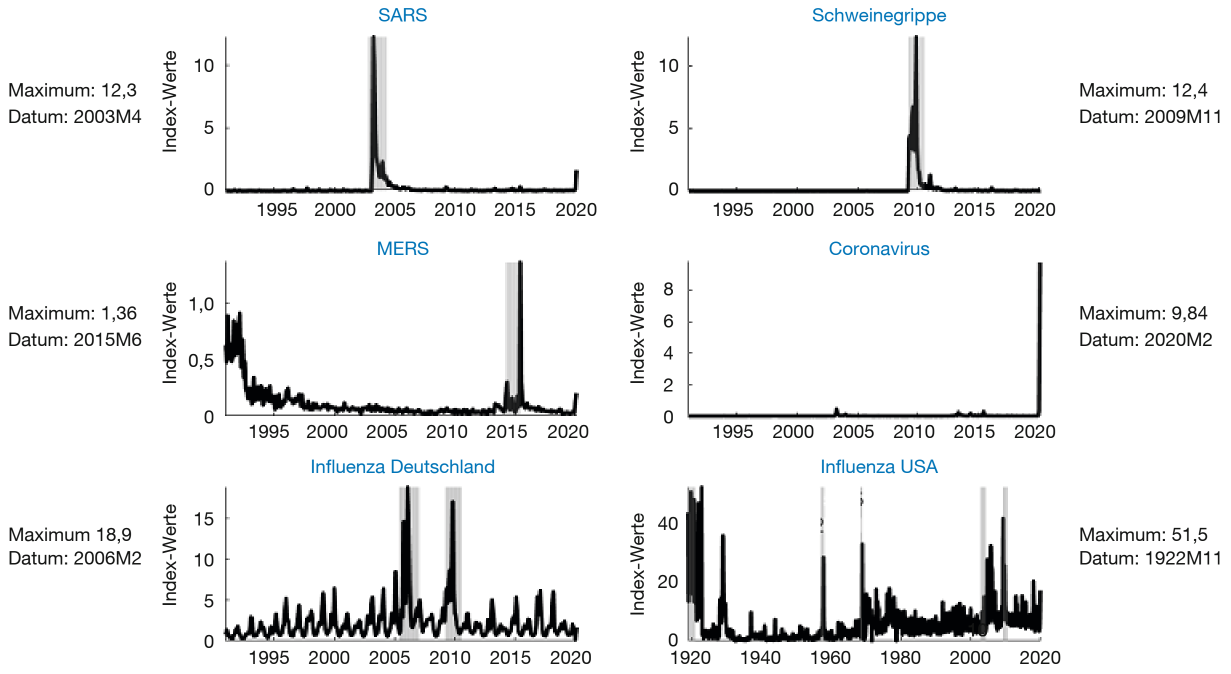

Quelle: Kholodilin und Rieth (2020a).

onsspielräume der Unternehmen ein. Hinzu kommt eine starke Verunsicherung sowohl bei Unternehmen als auch bei Haushalten über die wirtschaftlichen Rahmenbedingungen und den Verlauf der Pandemie. Dies führt zu einer deutlichen Zurückhaltung bei der Anschaffung von Investitions- und Konsumgütern und verstärkt die Rezession.

## Angebots- und Nachfrageschock kumulieren

Eine zentrale Frage für die konjunkturpolitische Bewertung der aktuellen Lage ist, in welchem Umfang angebotsseitige und nachfrageseitige Schocks kumulieren. Pandemieereignisse sind in der Wirtschaftsgeschichte selten – Lehren aus der Vergangenheit sind entsprechend schwer zu ziehen. Auswertungen auf Grundlage von Mediendaten zur Stärke epidemiologischer Ereignisse deuten allerdings darauf hin, dass diese sowohl auf der Angebots- als auch auf der Nachfrageseite zu starken Verwerfungen führen. Abbildung 1 zeigt historische Werte von Mediennennungen einzelner Epidemieereignisse wie Schweinegrippe, spanische Grippe oder Coronavirus in Relation zur Zahl der Artikel in dem jeweiligen Medienbeitrag. Diese Daten können für die Abschätzung der gesamtwirtschaftlichen Effekte, den Auswirkungen auf den Arbeitsmarkt oder auf die Preisentwicklung verwendet werden (Kholodilin und Rieth 2020a und 2020b).

Insgesamt zeigen diese Analysen, dass Epidemieereignisse kontraktiv und deflationär wirken. Sie haben erhebliche negative Effekte auf den weltweiten Handel, die Produktion, die Beschäftigung sowie die Entwicklung der Konsumentenpreise (vgl. Abbildung 2). Zudem sind die Folgen derartiger Ereignisse über einen längeren Zeitraum spürbar. Eine globale Erholung setzt erst nach etwa einem halben Jahr ein, ein Aufholen wirtschaftlicher Aktivitäten bleibt zögerlich. Zu ähnlichen Einschätzungen kommen auch Auswertungen zur aktuellen Krise auf Grundlage von Unternehmensbefragungen. Die Studien zeigen, dass die Preis- und Absatzerwartungen infolge der Corona-Pandemie erheblich gesunken sind (Balleer et al., 2020). Beide Analysen zeigen, dass geld- und fiskalpolitische Impulse notwendig sind, um die wirtschaftlichen Folgen der Corona-Krise zu mildern.

## Konjunkturprogramm ein kräftiger Impuls zur Stabilisierung

Die Bundesregierung hat ein Konjunkturpaket beschlossen, das einen erheblichen Impuls von insgesamt rund 130 Mrd. Euro zur Stabilisierung der Wirtschaftsleistung gibt. Dieser dürfte die wirtschaftliche Aktivität spürbar anregen. Schätzungen zeigen, dass die Stabilisierungswirkung in den Jahren 2020 und 2021 mit jeweils 1,3 Pro-

Abbildung 2
**Wirtschaftliche Folgen von Epidemien**
Prozentuale Abweichungen vom Niveau der Baselineprognose ohne Corona-Schock auf Grundlage eines VAR-Modells

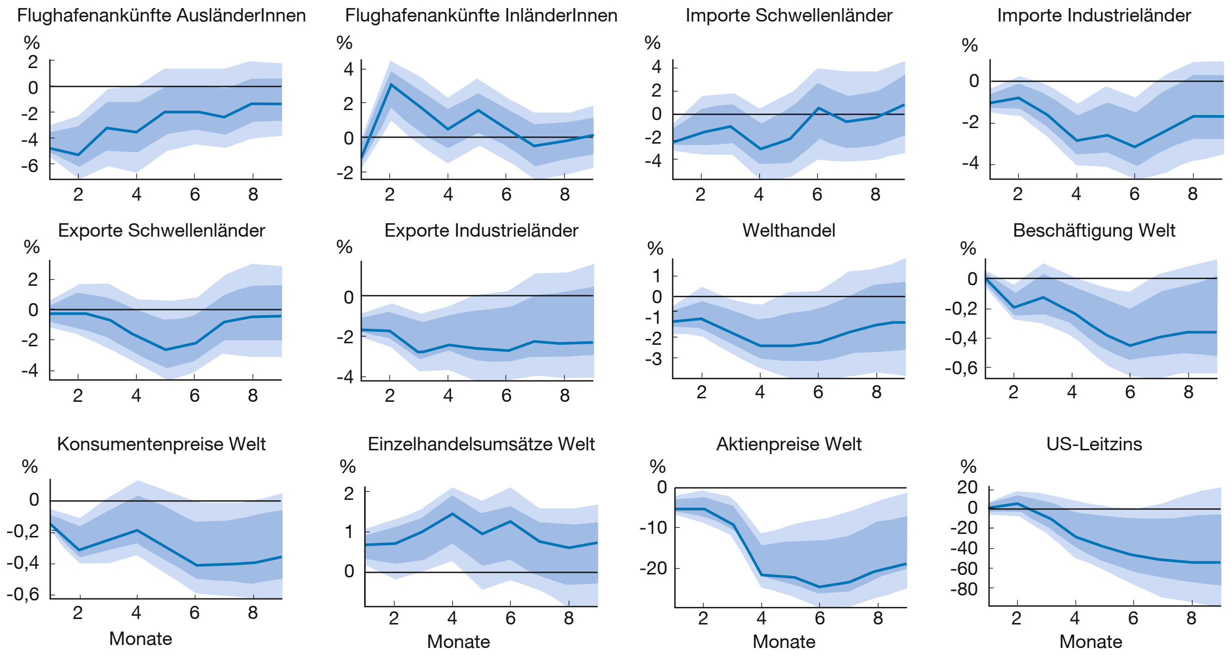

Quelle: Kholodilin und Rieth (2020b).

zentpunkten erheblich ist und mit den getroffenen Maßnahmen mehr als eine halbe Millionen Arbeitsplätze geschaffen bzw. erhalten werden (vgl. Tabelle 1).

Kern des Programms sind die zeitweise Absenkung der Mehrwertsteuer, eine Absenkung der EEGUmlage, ein Kinderbonus, die Anhebung des Entlastungsfreibetrags

Tabelle 1
**Maßnahmen und gesamtwirtschaftliche Effekte des Konjunkturprogramms**
in Mrd. Euro

| Maßnahmen | 2020 | 2021 | 2022 | 2022 | 2023 | 2024 | Gesamtvolumen |
|---|---|---|---|---|---|---|---|
| Investive Ausgaben | 11,4 | 15,1 | 6,3 | 3,3 | 2,3 | 1,0 | 39,3 |
| Konsumtive Ausgaben | 7,0 | 5,2 | 4,2 | 4,2 | 2,7 | 1,5 | 24,9 |
| Transfers und Subventionen | 14,3 | 13,2 | 9,0 | 3,7 | 2,4 | 1,3 | 43,9 |
| Steuermindereinnahmen | 33,0 | 1,0 | -7,0 | -5,0 | 0 | 0 | 22,0 |
| Finanzpolitische Konjunktur– und Wachstumsimpulse | 65,6 | 34,4 | 12,5 | 6,3 | 7,5 | 3,8 | 130,0 |

| Auswirkungen auf | 2021 | 2022 | 2022 | 2023 | 2024 | 2025 |
|---|---|---|---|---|---|---|
| Bruttoinlandsprodukt | 41,3 | 41,2 | 19,3 | 15,4 | 15,0 | 8,4 |
| Privater Konsum | 44,4 | 40,6 | 20,6 | 17,3 | 15,9 | 10,5 |
| Bruttoanlageinvestitionen | 21,9 | 23,7 | 9,0 | 5,7 | 6,3 | 2,6 |
| Handelsbilanz | -33,9 | -27,7 | -14,6 | -11,5 | -11,0 | -5,2 |
| Beschäftigung (in 1.000 Personen) | 108 | 196 | 153 | 99 | 86 | 65 |

Quelle: Abschätzungen auf Grundlage des DIW MAKRO-Modells, Michelsen et al. (2020).

für Alleinerziehende und die Deckelung der Sozialbeiträge. Diese Maßnahmen werden zusammen mehr als ein Drittel der Gesamtsumme kosten und dienen in erster Linie der Stärkung des privaten Verbrauchs. Darüber hinaus gibt es Punkte, die den Unternehmen bei der Bewältigung der Krisenfolgen helfen sollen: Zu nennen wären beispielsweise der erweiterte Verlustrücktrag, Kredite für gemeinnützige Organisationen und den Kulturbereich, Überbrückungshilfen und Beihilfen für die Unternehmen des Öffentlichen Personennahverkehrs (ÖPNV). Letztlich sind auch die Übernahmen kommunaler Ausgaben und Einnahmeausfälle aus der Gewerbesteuer ein Beitrag, um den Gebietskörperschaften an anderer Stelle – insbesondere bei den Investitionen – Handlungsspielräume zu verschaffen.

Ein weiterer großer Teil der vereinbarten Hilfen soll auch die Investitionstätigkeit anregen. Hierunter fallen einerseits die degressive Absetzung für Abnutzung (AfA) und die Aufstockung einer Vielzahl kleinerer, bereits existierender Förderprogramme wie die energetische Gebäudesanierung oder die steuerliche Forschungszulage. Zudem werden der Ausbau der Ganztagsbetreuung in Grundschulen und die Ausweitung der Kapazitäten in der Kinderbetreuung unterstützt. Diese Punkte werden überwiegend im Zukunftspaket zusammengefasst. Dieser Fokus auf die Investitionstätigkeit ist grundsätzlich zu begrüßen. Die Idee, an bestehende Förderprogramme anzuschließen, ist für eine schnelle Umsetzung hilfreich. Allerdings werden damit auch Chancen vertan, wichtige Impulse für eine strukturelle Veränderung der deutschen Wirtschaft zu setzen und ein höheres Potenzialwachstum zu erreichen. Dies betrifft beispielsweise Investitionen von Unternehmen, die Förderung von Innovationen, aber auch die Unterstützung von Entrepreneurship. Zudem gibt es langfristige Herausforderungen, die mit einer zu engen Fokussierung auf die kurzfristige Stabilisierung aus dem Blick zu geraten drohen.

## Notwendigkeit eines Zukunftspakets für die wirtschaftliche Transformation

Der Fokus in der ersten Phase der Krise lag auf der kurzfristigen Stabilisierung der Wirtschaft durch massive Unterstützung von Unternehmen und des privaten Konsums mit dem primären Ziel, Unternehmensinsolvenzen und einen starken Anstieg der Arbeitslosigkeit möglichst zu vermeiden. Diese Politik war klug und auch effektiv, um Schlimmeres zu verhindern. In der zweiten Phase der Pandemie muss jedoch nun das Hauptaugenmerk auf der Transformation der deutschen Wirtschaft liegen. Wenn Konjunkturprogramm, Wirtschaftsstabilisierungsfonds und andere stabilisierende Maßnahmen zu einer Zementierung alter Strukturen führen, dann ist die Gefahr groß, dass die deutsche Wirtschaft langfristig an Wettbewerbsfähigkeit verliert und der Wirtschaftsstandort Deutschland gefährdet wird. Daher sollte die Wirtschaftspolitik nun in den kommenden Monaten und Jahren oberste Priorität den Zukunftsinvestitionen und der Transformation hin zu einer innovativen, digitalen und klimaneutralen Wirtschaft legen. Hierfür hat das DIW Berlin, wie auch andere Institute, einen konkreten Vorschlag gemacht, wie ein solches Zukunftspaket aussehen sollte.

Tabelle 2
**Maßnahmen eines Investitions- und Transformationspakets bis 2030 insgesamt**

| Maßnahmen | Mrd. Euro |
|---|---|
| Degressive Afa und Sofortabschreibungen | 6,4 |
| Investitionsfonds | 44,0 |
| Innovationsförderung | 3,0 |
| Gründungsförderung Investitionen | 9,0 |
| Energetische Gebäudesanierung (KfW) | 9,0 |
| Solarprämie (100.000 Anlagen) | 1,0 |
| Fahrradinfrastruktur | 1,5 |
| Schienenverkehr | 15,0 |
| ÖPNV | 5,0 |
| Umstiegsprämie für Fahrräder und ÖPNV | 7,5 |
| Investitionen in Ganztagsangebote für Grundschulkinder | 9,6 |
| Betriebskosten Ganztagsangebote für Grundschulkinder | 25,6 |
| Kommunale Altschulden (Bundes- und Länderanteil) | 43,0 |
| Kommunalinvestitionsfonds | 33,0 |
| Klimaneutrale Produktions- und Recyclingprozesse | 8,7 |
| Schutzschirm für Kommunen (Wegfall Gewerbesteuer) | 14,0 |
| Summe | 235,3 |

Quelle: Belitz et al. (2020).

Deutschland hat seit geraumer Zeit ein großes Investitionsproblem. Vielfach wurde auf die erheblichen Investitionsbedarfe angesichts des fortschreitenden demografischen Wandels, der notwendigen Dekarbonisierung, im Bereich der Bildung, der Digitalisierung oder der kommunalen Infrastruktur hingewiesen (Gornig et al., 2015; Gornig, 2019; Gornig und Michelsen, 2017; Fratzscher, 2016; Belitz und Gornig, 2019; Dullien et al., 2019; Clemens, 2019; Fratzscher et al., 2016; Spieß, 2013; Blazejczak et al., 2013; Kunert und Link, 2013). Auch droht die Innovationskraft in der Krise verloren zu gehen – insbesondere wenn die Investitionen rückläufig sind. In der derzeitigen konjunkturellen Lage ist es daher sinnvoll, diese Investitionsbedarfe anzugehen, die Konjunktur zu stabilisieren

Tabelle 3

**Maßnahmen und makroökonomische Auswirkungen eines Investitions- und Transformationspakets p. a.**

in Mrd. Euro (real)

| Maßnahmen | 2020 | 2021 | 2022 | 2023 | 2024 | 2025 | 2026 bis 2030 | Gesamt-volumen |
|---|---|---|---|---|---|---|---|---|
| Öffentliche Investitionen | 12,8 | 13,1 | 9,9 | 9,6 | 8,3 | 7,4 | 32,2 | 93,3 |
| Staatskonsum | 6,1 | 7,2 | 6,2 | 5,3 | 5,2 | 4,9 | 25,9 | 60,8 |
| Transfers und Subventionen | 10,7 | 8,8 | 4,5 | 2,5 | 2,5 | 2,4 | 6,8 | 38,2 |
| Finanzpolitische Konjunktur- und Wachstumsimpulse | 29,6 | 29,0 | 20,6 | 17,4 | 16,1 | 14,6 | 64,9 | 192,3 |
| Hilfsfonds Kommunen (Altschulden) | 43,0 | | | | | | | |
| Gesamtpaket | 72,6 | 29,0 | 20,6 | 17,4 | 16,1 | 14,6 | 64,9 | 235,3 |

| Makroökonomische Auswirkungen | 2020 | 2021 | 2022 | 2023 | 2024 | 2025 |
|---|---|---|---|---|---|---|
| Bruttoinlandsprodukt | 24,6 | 30,5 | 23,5 | 21,3 | 20,6 | 18,9 |
| Privater Konsum | 23,0 | 27,8 | 23,3 | 21,5 | 20,8 | 19,5 |
| Bruttoanlageinvestitionen | 15,6 | 20,0 | 13,1 | 11,9 | 11,6 | 10,5 |
| Handelsbilanz | -19,1 | -23,8 | -18,8 | -17,0 | -16,6 | -15,5 |
| Beschäftigung (in 1.000 Personen) | 65 | 126 | 116 | 93 | 85 | 78 |

Quelle: Abschätzungen auf Grundlage des DIW MAKRO-Modells, Belitz et al. (2020).

und damit auch einen notwendigen strukturellen Wandel anzustoßen. Bekannt ist, dass die Multiplikatoren öffentlicher Ausgaben in konjunkturellen Krisen besonders groß sind – gerade im Niedrigzinsumfeld. Öffentliche Investitionen sind zudem Anstoß für die private Investitionstätigkeit (Clemens et al., 2019). Entsprechende finanzpolitische Spielräume sind trotz der schweren Krise und der damit verbundenen Belastung der öffentlichen Haushalte nach wie vor gegeben. Die Zinsen von Bundesanleihen sind mit langen Laufzeiten negativ. Zudem haben Investitionen in Krisenzeiten eine besonders hohe Rendite und finanzieren sich durch zukünftig höheres Wachstum in weiten Teilen selbst.

Angesichts dessen ist es nun wichtig, neben dem beschlossenen Konjunkturprogramm ein weiteres Zukunftsprogramm anzustoßen, das die Transformationsprozesse befördert und dadurch das Wachstumspotenzial stärkt. Ein über mehrere Jahre gestrecktes, ambitioniertes Programm stabilisiert die Erwartungen und stärkt damit ebenfalls die Investitionsneigung von Unternehmen und Haushalten heute. Entsprechende Vorschläge wurden bereits unterbreitet. Diese zielen auf die Stärkung privater Investitionen mit Hilfe eines europäischen Fonds, Impulsen im Bereich der Forschung und Entwicklung, die Förderung von Entrepreneurship, Klima- und Umweltschutz, Bildung, Digitalisierung und auf die Stärkung kommunaler Investitionen ab und umfasst ein Gesamtvolumen von rund 235 Mrd. Euro gestreckt über die kommenden zehn Jahre (Belitz et al., 2020, vgl. Tabellen 2 und 3).

Insgesamt dürfte das Programm über die Gesamtdauer von zehn Jahren bis 2030 rund 890.000 neue Jobs schaffen und damit den prognostizierten Anstieg der Arbeitslosigkeit in der Corona-Krise überkompensieren können. Allerdings werden für viele der Maßnahmen Fachkräfte benötigt. Insbesondere der angeregte Ausbau von Ganztagsschulen sowie die notwendige betriebliche Ausstattung der Kitas und Schulen mit Personal dürfte hier eine zentrale Rolle spielen, um allen Eltern die Möglichkeit zu geben, guten Gewissens in Vollzeit zu arbeiten.

Auch die Rückwirkungen des Investitionsprogramms auf die Staatfinanzen können abgeschätzt werden. Bis 2030 können zusätzliche Einnahmen aus dem Programm von rund 92 Mrd. Euro die Ausgaben teilweise finanzieren. Denn insbesondere Steuereinnahmen und Sozialbeiträge dürften durch Produktivitätsgewinne und höhere Löhne, Beschäftigungs- und Gewinndynamik zunehmen. Der Schuldenstand in % des nominalen Bruttoinlandsprodukts dürfte trotz des erheblichen Impulses lediglich um vier Prozentpunkte ansteigen.

### Konjunkturprogramm durch Zukunftspaket ergänzen

Die Corona-Krise trifft die Wirtschaft sowohl von der Angebots- als auch von der Nachfrageseite. Die Europäische Zentralbank hat weitreichende Maßnahmen beschlossen, um die Konjunktur zu stabilisieren. Sie stößt in dieser Krise allerdings an ihre Grenzen. Angesichts dessen sind finanzpolitische Maßnahmen notwendig. Deutschland verfügt über entsprechende haushaltspolitische Spielräume, um die Folgen der Krise zu reduzieren und eine Erholung finanzpolitisch zu unterstützen. Das Konjunkturpaket der Bundesregierung ist in dieser Hinsicht ein wichtiger und in weiten Teilen sinnvoller Schritt (Bach und Michelsen, 2020).

Damit dieses Konjunkturpaket nicht nur kurzfristig, sondern auch langfristig wirkt und damit es nicht kontraproduktiv wird und alte Strukturen zementiert, muss es mittelfristig durch ein kluges Zukunftspaket ergänzt werden. Dies sollte ein Investitionsprogramm für nachhaltige, digitale Technologien, eine verbesserte digitale und Energieinfrastruktur, eine deutliche Verbesserung des Bildungssystems und die Förderung von jungen Unternehmen enthalten. Ansonsten ist die Gefahr groß, dass das Konjunkturprogramm verpufft und die wirtschaftliche Erholung in den kommenden Jahren lähmen könnte.

Prof. Dr. Marcel Fratzscher ist Hochschullehrer an der Humboldt-Universität zu Berlin und Präsident des Deutschen Instituts für Wirtschaftsforschung (DIW) in Berlin.

Dr. Claus Michelsen leitet die Abteilung Konjunkturpolitik am Deutschen Institut für Wirtschaftsforschung in Berlin (DIW Berlin).


Literatur

Bach, S. und C. Michelsen (2020), Stellungnahme im Rahmen der öffentlichen Anhörung des Finanzausschusses des Deutschen Bundestages, 22. Juni, Zweites Corona-Steuerhilfegesetz ist eine richtige Antwort auf die Corona-Krise.
Balleer, A. et al. (2020), Demand or Supply? Price Adjustment during the Covid-19 Pandemic, CESifo Working Paper, Nr. 8394.
Belitz H Gornig M Deutsche Wirtschaft muss mehr in ihr Wissenskapital investieren DIW Wochenbericht 2019 86 31 527 534
Belitz H Mit Investitionen und Innovationen aus der Corona-Krise DIW Wochenbericht 2020 87 24 442 451
Blazejczak J Energiewende erfordert hohe Investitionen DIW Wochenbericht 2013 80 26 19 30
Clemens M Öffentliche Finanzen: Haushaltsspielräume verflüchtigen sich nach und nach — Investitionsprogramm wäre sinnvoll DIW Wochenbericht 2019 86 50 952 960
Clemens M Goerge M Michelsen C Öffentliche Investitionen sind wichtige Voraussetzung für privatwirtschaftliche Aktivität DIW Wochenbericht 2019 86 31 537 543
Dullien S Konjunkturpolitik in der Krise Wirtschaftsdienst 2019 99 11 747 768 10.1007/s10273-019-2526-5
Fratzscher, M. (2016), Stärkung von Investitionen in Deutschland. Stellungnahme der Expertenkommission im Auftrag des Bundesministers für Wirtschaft und Energie, Sigmar Gabriel.
Fratzscher M Gornig M Schiersch A Investitionsschwäche der Unternehmen schafft Handlungsbedarf DIW Wochenbericht 2016 83 15 275 280
Gornig M Infrastrukturinvestitionen statt Subventionen Wirtschaftsdienst 2019 99 1 44 48 10.1007/s10273-019-2431-y
Gornig M Michelsen C Kommunale Investitionsschwäche: Engpässe bei Planungs-und Baukapazitäten bremsen Städte und Gemeinden aus DIW Wochenbericht 2017 84 11 211 219
Gornig M Michelsen C van Deuverden K Kommunale Infrastruktur fährt auf Verschleiß DIW Wochenbericht 2015 82 43 1023 1030
Kholodilin, K. und M. Rieth (2020a), Viral Shocks to the World Economy, DIW Discussion Paper, Nr. 1861.
Kholodilin, K. A. und M. Rieth (2020b), Medienbasierter Index zeigt: Epidemien bringen in der Regel dauerhafte wirtschaftliche Einbußen mit sich, DIW aktuell, (32).
Kunert U Link H Verkehrsinfrastruktur: Substanzerhaltung erfordert deutlich höhere Investitionen DIW Wochenbericht 2013 80 26 32 38
Michelsen C Clemens M Hanisch M Junker S Kholodilin K A Pagenhardt L Schlaak T Deutsche Wirtschaft: Schleppende Erholung nach tiefem Fall: Grundlinien der Wirtschaftsentwicklung im Sommer 2020 DIW Wochenbericht 2020 87 24 420 436
Spieß K Investitionen in Bildung: frühkindlicher Bereich hat großes Potential DIW Wochenbericht 2013 80 26 40 47
